# pCLE detects mucosal neoplastic vascular pattern in gastric linitis plastica

**DOI:** 10.1007/s10238-022-00843-y

**Published:** 2022-06-01

**Authors:** Mara Fornasarig, Alessandra Capuano, Stefania Maiero, Eliana Pivetta, Vincenzo Canzonieri, Claudio Belluco, Maurizio Mongiat, Renato Cannizzaro, Paola Spessotto

**Affiliations:** 1 Oncological Gastroenterology - Centro di Riferimento Oncologico di Aviano (CRO), IRCCS, Aviano, Italy; 2 Molecular Oncology - Centro di Riferimento Oncologico di Aviano (CRO), IRCCS, Aviano, Italy; 3 Pathology - Centro di Riferimento Oncologico di Aviano (CRO), IRCCS, Aviano, Italy; 4 Department of Medical, Surgical and Health Sciences, University of Trieste, 34127 Trieste, Italy; 5 Surgical Oncology - Centro di Riferimento Oncologico di Aviano (CRO), IRCCS, Aviano, Italy

**Keywords:** Confocal endomicroscopy, Vessel alterations, Linitis plastica, Angiogenesis

## Abstract

Linitis plastica (LP) is a very aggressive and rare carcinoma with a scirrhous stroma that affects the submucosal and muscular layers of the stomach even without mucosal alterations. Lack of timely diagnosis is a crucial problem related to its prognosis and treatment. In this study, we investigated the LP-associated vascular pattern as a possible means to improve the diagnosis of these patients. During standard endoscopy, mucosal architecture, tortuosity and enlargement of vessels, as well as the presence of vascular leakage and efficiency of the blood flow were assessed in six LP patients using probe-based Confocal Laser Endomicroscopy (pCLE). In all LP patients, we detected abnormal changes in vasculature. The aberrant features of the vascular network were common to all LP patients examined and consisted of vessel enlargement, tortuosity, and leakage associated with the affected submucosal layer. This is the first study to highlight the presence of marked vascularization associated with LP, characterized by the presence of abnormal and non-functional vessels, similar to what is observed in neoplastic tissues. Therefore, the analysis of LP by pCLE may provide a new endoscopic approach and strategy to better define these patients.

## Introduction

Linitis plastica (LP) is an aggressive and rare carcinoma with a scirrhous stroma involving the submucosal and muscular layers of the stomach [1]. The spread pattern of LP tumors primarily involves the submucosa and muscularis propria of the stomach, while mucosal involvement is irregular. Late neoplastic infiltration of the mucosa may delay pathologic diagnosis and worsen prognosis. In fact, endoscopic biopsies are not suitable for the diagnosis of neoplasia in 30% of cases [2]; moreover, the gastric mucosa may have the specific features of atrophic gastritis, leading to misdiagnosis. Reduction in distensibility of the gastric wall is the most important endoscopic feature of LP, but usually represents a late event. Endoscopic ultrasound (EUS) specifically provides the ability to assess the thickness of the gastric wall and evaluate the different layers of the stomach and the depth of the malignancy [3].

The probe-based Confocal Laser Endomicroscopy (pCLE) enables in vivo analysis of tissue microarchitecture and precise identification of areas suitable for biopsy sampling by enhancing contrast and magnification of the diseased tissues at the cellular level [4–7]. High-resolution confocal imaging is achieved by i.v. injection of fluorescein and allows endoscopists to visualize cellular and subcellular structures as well as capillaries and flowing erythrocytes in vivo [8]. Thus, the technique provides not only structural but also functional information as the leakage of the dye from the less efficient vessels can be detected and measured. This technique has also been used in a few studies to objectively assess microvessel density at different neoplastic stages [9–11] and angiogenic status of gastrointestinal cancers [12–14]. Indeed, pCLE has been shown not only to be a useful tool but also a valid technique for the evaluation of vascular changes in gastric cancer [14, 15].

With the aim of developing new approaches to improve LP diagnosis, in this study, we used the pCLE technique for the first time to determine whether these lesions are also characterize by aberrant vasculature similar to gastric cancer.

## Materials and methods

### Patients

For this study, we consecutively enrolled six patients (100% female, mean age 66) who underwent endoscopy for diagnostic work up in 2019–2020 at the Oncological Gastroenterology Unit of the CRO-IRCCS, National Cancer Institute of Aviano (PN), Italy. Based on biopsy specimens, patients (“1-6LP”) were diagnosed negative for gastric cancer, although the endoscopic features were macroscopically suggestive for LP. pCLE was used to guide biopsy sampling and define vascular architecture. All patients consented to endomicroscopic pCLE analyses by signing the informed consent form. The methods used were in accordance with the standards of the WMA Declaration of Helsinki. The study was approved by the Institutional Board of the CRO-IRCCS (IRB no. CRO-2014-03).

### Endoscopy procedures and pCLE analyses

The pCLE analyses were performed using a GastroFlex UHD probe (Cellvizio, Mauna Kea Technology, Paris, France) during gastroscopy (Olympus series 180) and immediately before endoscopic ultrasonography (Olympus series 160) as previously described [12, 13]. After careful inspection of the gastric mucosa by HD white light and NBI, images were obtained of the mucosa of the antrum, angulus, antrum/corpus border, small and great curvature of the body and cardias followed by conventional biopsy collection by macrobiopsy (COOK Medical, Ireland) at the end of the examination. Images were acquired within the first 10 min after i.v. injection of fluorescein (3 ml of 10% solution). pCLE images were acquired at 12 frames per second to ensure high video quality and direct visualization at the level of individual erythrocytes. pCLE acquisitions were performed for at least 3 min, resulting in real-time imaging of more than 2000 frames. Mucosal architecture, presence of leakage, vessel tortuosity and enlargement and efficiency of blood flow were assessed using the Cannizzaro-Spessotto score as previously described [14]. Images were stored digitally and reviewed using a dedicated software package (Cellvizio Viewer, Mauna Kea Technologies) by a highly experienced investigator who was blinded to any clinical, endoscopic or histopathological information.

### Histology

Gastric cancer was classified according to the Lauren classification [16], and the disease stage was assessed according to the TNM criteria.

## Results

**Fig. 1:**
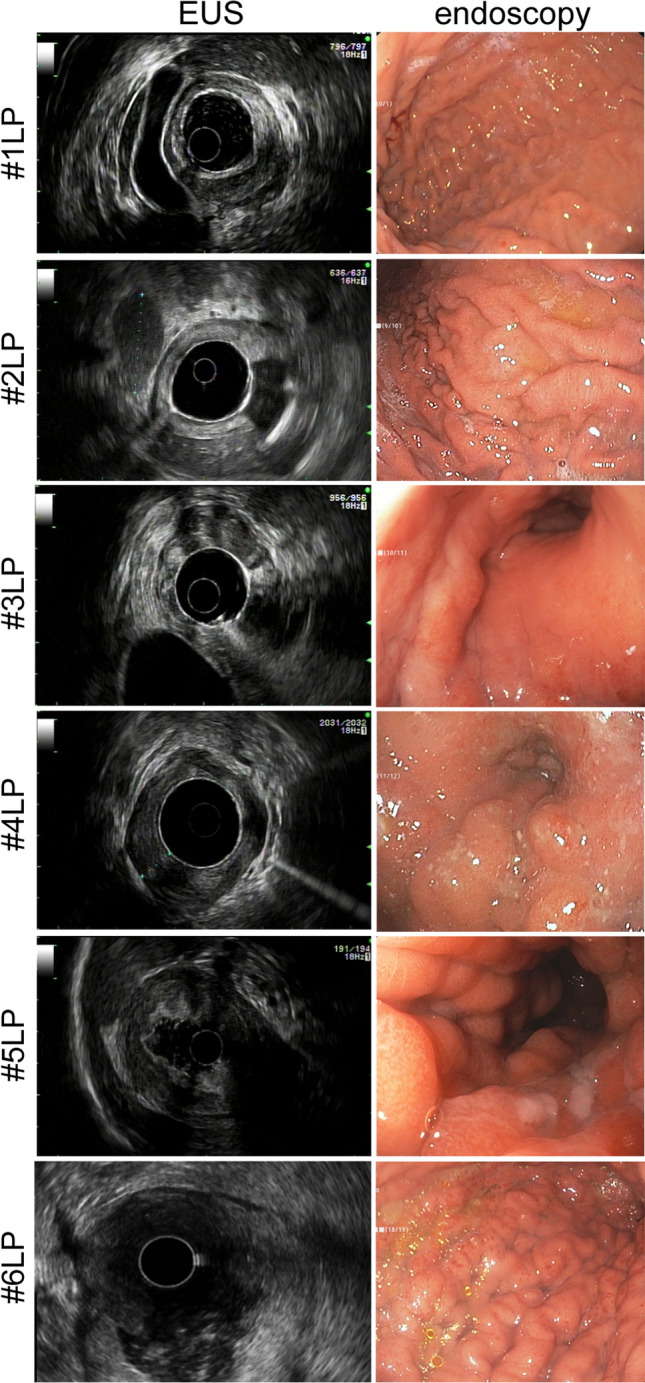
Representative images of LP patients obtained during EUS and endoscopic evaluation

pCLE could not detect neoplastic features in the gastric mucosa of all examined LP patients. The gastric pit pattern showed reduced but regular pits with dilated openings as observed in atrophic gastritis [11]. Accordingly, cancerous lesions could not be diagnosed based on the analyses of the endoscopic biopsies. Representative images of the endoscopic and EUS analyses are shown in Fig. 1. Endoscopy showed poor expansion of the gastric walls and huge, swollen, straight, furrowed, and crossed gastric folds. EUS showed blurred layers replaced by hypoechogenic thickening of the gastric wall. Patient #2LP was found to have a large metastatic lymph node (45 mm, larger diameter). The pathologic diagnosis of LP was made by intramural biopsies during laparoscopy in 5 cases and by fine-needle aspiration of a peri-gastric lymph node by EUS in 1 case.

**Fig. 2:**
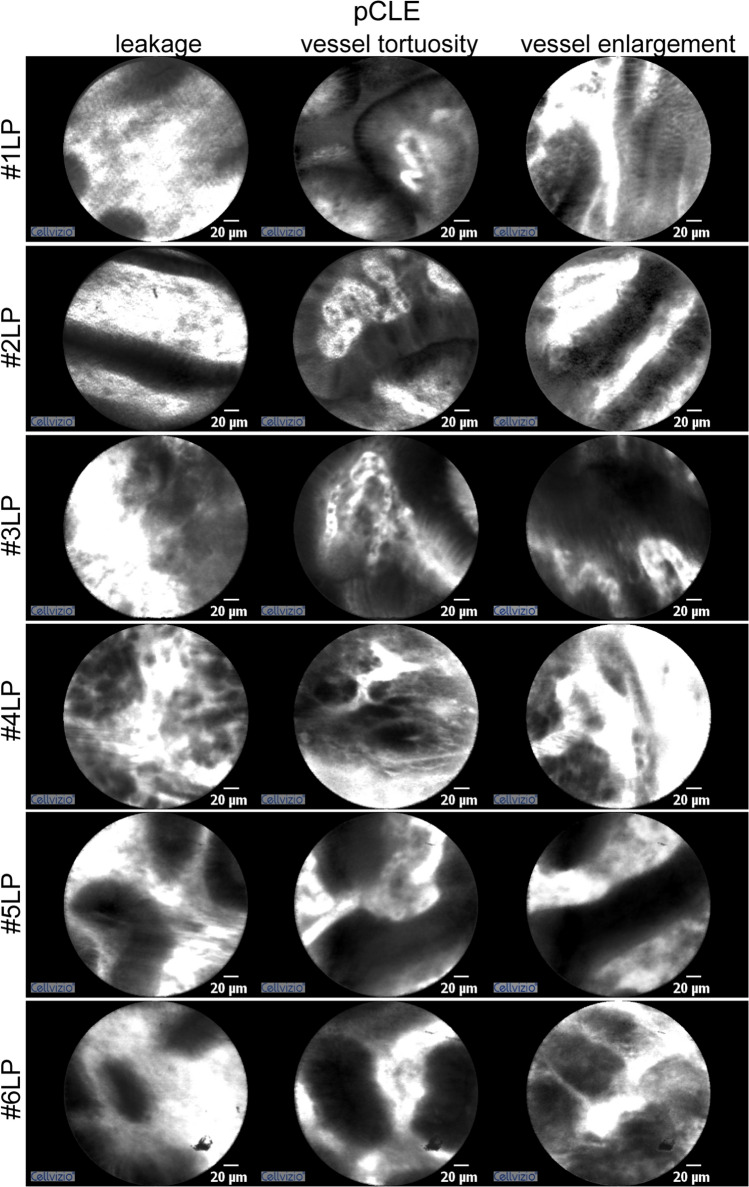
Representative images of LP patients obtained using pCLE. The vascular pattern in all LP patients shows the presence of leakage, vessel tortuosity and vessel enlargement

Although confocal endomicroscopy excluded neoplastic mucosal infiltration, pCLE was useful in revealing the features of vascular architecture in this type of gastric cancer. The vessel architecture was abnormal and mainly consisted of enlarged, distorted and highly permeable microvessels. Figure 2 clearly shows the presence of irregular, tortuous, large-sized vessels, also characterized by extensive leakage into the gastric mucosa. The peculiar morphology and patchy conformation of these capillaries are probably due to the increasing thickness and subsequent compression of the mucosal layer. Accordingly, the tortuous vessels discovered in LP patients did not appear to be regularly distributed, as is generally the case in atrophic mucosa [13]. The efficiency of the vessels in relation to blood flow was also studied. However, LP-associated vessels were not characterized by aberrant blood flow. Interestingly, all the patients studied showed the same vascular alterations, i.e. leakage, vessel tortuosity and enlargement, which were always present qualitatively and quantitatively.

## Discussion

The lack of timely diagnosis is a crucial problem related to the prognosis and treatment of LP. Because of their peculiar spread pattern, LP tumors occasionally cannot be distinguished from atrophic gastritis on endoscopy. Endoscopy of the upper gastrointestinal tract reveals only the mucosal layer, and superficial biopsies may be negative in this case. Because tumor cells generally migrate throughout the submucosa without seriously affecting the gastric mucosa, it is difficult at an early stage to detect LP-derived cancer cells by gastrointestinal series or conventional endoscopic biopsies. Indeed, analyses of endoscopic biopsies in all LP patients who participated in this study were inadequate to correctly diagnose the disease. The use of pCLE, a well-established endoscopic tool to improve diagnosis in gastrointestinal diseases [6], was helpful to exclude the presence of mucosal infiltration by cancer cells.

Recently, we thoroughly investigated the vascular features of locally advanced gastric cancer by evaluating the use of pCLE for the analysis of intratumoral angiogenesis and demonstrated that the functional and structural angiogenic parameters of the tumor blood network were fully detectable with this innovative endoscopic technique [13]. The vascular alterations in gastric cancer were mainly characterized by leakage and the presence of tortuous and large vessels. Defects in blood flow were rarely detected [13]. In this study, we described for the first time the vascular aberrations associated with the LP mucosa and showed that in all patients, the blood vessels were clearly visible and could be well resolved by pCLE, which allowed to distinguish the typical aberrant features characterizing the tumor-associated blood vessels, as previously described in locally advanced gastric cancer [12, 13]. Indeed, LP-associated gastric mucosa was characterized by abundant vascularization, although the vessels were predominantly abnormal and non-functional. In fact, all patients consistently exhibited vascular leakage, tortuosity and enlargement. The tortuous vessels in LP patients were not regular but scattered and unevenly distributed. It can be speculated that the reactive fibrosis characterizing the affected submucosal layer and the resulting mechanical compression favor the formation of tortuous, enlarged, leaky and non-functional vessels.

A major limitation of this study is that it is a qualitative analysis performed on a small number of patients, although this disease is quite rare, especially in Western countries. On the other hand, we believe that the strength of this study lies in the innovative use of this endoscopic technique, which, unlike other techniques currently used, can provide not only structural but also functional information about the tissue. Nevertheless, to our knowledge, this is the first report showing the use of the pCLE technique for the analysis of LP patients, as well as the first time that a thorough analysis of the LP-associated vasculature is outlined. Given the promising results of this study, we envision that the use of pCLE may open new perspectives not only to improve diagnosis but also to better understand how vascular characteristics may influence the biological behavior of gastric disease.
